# Association between empirically derived dietary patterns with blood lipids, fasting blood glucose and blood pressure in adults - the India migration study

**DOI:** 10.1186/s12937-018-0327-0

**Published:** 2018-02-08

**Authors:** Krithiga Shridhar, Ambika Satija, Preet K. Dhillon, Sutapa Agrawal, Ruby Gupta, Liza Bowen, Sanjay Kinra, A. V. Bharathi, D. Prabhakaran, K. Srinath Reddy, Shah Ebrahim, for the Indian Migration Study group

**Affiliations:** 1 Centre for Chronic Conditions and Injuries, Public Health Foundation of India, Gurgaon, Haryana India; 2 Harvard T. H. Chan School of Public Health, Boston, USA; 3 London School of Hygiene and Tropical Medicine, London, UK; 4 Just Right Obesity Clinic, Bangalore, India; 5 Centre for Chronic Disease Control, Gurgaon, Haryana India; 6 Public Health Foundation of India, New Delhi, India

**Keywords:** Lipids, Dietary patterns, Animal food, Fasting glucose, Indian migration study, India

## Abstract

**Background:**

Dietary patterns (DPs) in India are heterogenous. To date, data on association of indigenous DPs in India with risk factors of nutrition-related noncommunicable diseases (cardiovascular disease and diabetes), leading causes of premature death and disability, are limited. We aimed to evaluate the associations of empirically-derived DPs with blood lipids, fasting glucose and blood pressure levels in an adult Indian population recruited across four geographical regions of India.

**Methods:**

We used cross-sectional data from the Indian Migration Study (2005–2007). Study participants included urban migrants, their rural siblings and urban residents and their urban siblings from Lucknow, Nagpur, Hyderabad and Bangalore (*n* = 7067, mean age 40.8 yrs). Information on diet (validated interviewer-administered, 184-item semi-quantitative food frequency questionnaire), tobacco consumption, alcohol intake, physical activity, medical history, as well as anthropometric measurements were collected. Fasting-blood samples were collected for estimation of blood lipids and glucose. Principal component analysis (PCA) was used to identify major DPs based on eigenvalue> 1 and component interpretability. Robust standard error multivariable linear regression models were used to investigate the association of DPs (tertiles) with total cholesterol (TC), low density lipoprotein-cholesterol (LDL-C), high density lipoprotein-cholesterol (HDL-C), triglycerides, fasting-blood glucose (FBG), systolic and diastolic blood pressure (SBP and DBP) levels.

**Results:**

Three major DPs were identified: ‘cereal-savoury’ (cooked grains, rice/rice-based dishes, snacks, condiments, soups, nuts), ‘fruit-vegetable-sweets-snacks’ (Western cereals, vegetables, fruit, fruit juices, cooked milk products, snacks, sugars, sweets) and ‘animal food’ (red meat, poultry, fish/seafood, eggs) patterns. High intake of the ‘animal food’ pattern was positively associated with levels of TC (β = 0.10 mmol/L; 95% CI: 0.02, 0.17 mmol/L; p-trend = 0.013); LDL-C (β = 0.07 mmol/L; 95% CI: 0.004, 0.14 mmol/L; p-trend = 0.041); HDL-C (β = 0.02 mmol/L; 95% CI: 0.004, 0.04 mmol/L; p-trend = 0.016), FBG: (β = 0.09 mmol/L; 95% CI: 0.01, 0.16 mmol/L; p-trend = 0.021) SBP (β = 1.2 mm/Hg; 95% CI: 0.1, 2.3 mm/Hg; p-trend = 0.032); DBP: (β = 0.9 mm/Hg; 95% CI: 0.2, 1.5 mm/Hg; p-trend = 0.013). The ‘cereal-savoury’ and ‘fruit-vegetable-sweets-snacks’ patterns showed no association with any parameter except for a positive association with diastolic blood pressure for high intake of ‘fruits-vegetables-sweets-snacks’ pattern.

**Conclusion:**

Our results indicate positive associations of the ‘animal food’ pattern with cardio-metabolic risk factors in India. Further longitudinal assessments of dietary patterns in India are required to validate the findings.

**Electronic supplementary material:**

The online version of this article (10.1186/s12937-018-0327-0) contains supplementary material, which is available to authorized users.

## Background

Cardiovascular disease (CVD) and type-II diabetes are major noncommunicable diseases (NCDs) accounting for 33.9% of total deaths [1] and 15.7% of all disability-adjusted life years-(DALY’s) [2] in 2013 worldwide. Over three-quarters of deaths due to CVD and diabetes occur in low-middle income countries (LMICs) [3] where the probability of dying between ages 30 and 70 years due to NCD is greater than in developed countries (25–30% vs. 10–15%) [3]. Diet is a key modifiable determinant of CVD and type-II diabetes, and potentially influences risk of developing these diseases by modulating blood levels of lipids and fasting glucose as well as blood pressure, important proximal risk factors of CVD and type-II diabetes [4–8].

‘Dietary patterns’ describe the overall diet; i.e. the varied combinations of foods and nutrients consumed in totality by individuals [9, 10]. Conventionally, nutritional epidemiological studies evaluated associations of individual food components or nutrients with several health outcomes. ‘Dietary pattern’ studies are relatively a recent approach in nutritional epidemiology studying the cumulative influence of composite diet as a ‘whole’ on different health outcomes. This approach can better account for any residual confounding by other components or nutrients of diet, and also provides useful evidence for more practical and appropriate dietary recommendations that are likely to succeed in real circumstances [9, 10].

A variety of dietary patterns (DPs) have been evaluated for their effects on CVD risk and mortality and diabetes risk including Dietary Approaches to Stop Hypertension (DASH), prudent and vegetarian diets, as well as regional diets such as the Mediterranean diet, Japanese diet and ‘Western’ diet [4–8]. Appropriate dietary recommendations are frequently updated based on this evidence [11, 12]. Dietary and culinary cultures are heterogenous in India and there is continued high adherence to indigenous food patterns [13, 14] in spite of the nutrition transition [15]. Evidence from India regarding the role of different dietary patterns on blood lipids, fasting glucose and blood pressure levels is critical given rising death and disability due to cardiovascular disease (leading at 53% increase in deaths and 41.5% increase in pre-mature deaths recorded between years 2005 and 2016) and diabetes (9% increase in disability between years 2005 and 2016) [16]. Diet and associated high blood pressure, fasting glucose and total cholesterol are cited as the leading risk factors of NCDs [16]. However, studies based on Indian dietary patterns are limited to date [9, 10].

We examined the associations of empirically derived indigenous dietary patterns with cardio-metabolic risk factors i.e., blood lipids, fasting blood glucose and blood pressure levels in rural and urban Indians recruited across four geographic regions of the country [Lucknow, North India; Nagpur, Central India; Hyderabad, South-central India and Bangalore, South India] using data from the Indian Migration Study [17, 18].

## Methods

### Study design and participants

Cross-sectional data from the Indian Migration Study (2005–2007) have been used for this study. The Indian Migration Study (IMS) is a sib-pair study nested within the larger Cardiovascular Disease Risk Factor Study in industrial populations from 10 companies across India [19] with details of the study design and methods reported earlier [17, 18]. In brief, the IMS was carried out in factory settings located in four cities from northern (Lucknow), central (Nagpur), south-central (Hyderabad), and southern (Bangalore) India. Factory workers, who had migrated from rural to urban areas (mean duration of migration 20 years ±5.4SD) and their co-resident spouses, along with a 25% random sample of urban non-migrants and their co-resident spouses were invited to participate in the study. Each migrant and non-migrant participant was asked to identify a sibling residing in a rural or urban area respectively, preferably of the same gender and similar age, who was then again invited to participate. This resulted in drawing of rural and urban dwelling siblings from 18 states across India. Of the 7594 migrant and non-migrant factory workers and their co-resident spouses and rural or urban siblings eligible for the study, 7102 (94%) agreed to complete the clinical examination. The final sample included 3537 sib-pairs (7074 participants) who completed the field work and 7067 respondents were included in the analyses as seven participants did not complete their clinical examination and/or some section of questionnaires.

### Description of predictor variables

An interviewer–administered questionnaire was used to collect information on diet, tobacco consumption, alcohol intake, physical activity and medical history in the local language. Anthropometric and physical measurements and fasting blood samples for measurement of blood lipid profiles and glucose levels were collected from participants. Standard of Living Index (SLI) was derived using a sub-set of questions on socio-economic position such as quality of house (kutcha (low quality)/semi-pucca (partly low quality)/pucca (high quality)), toilet facilities, land ownership, sources of lighting and drinking water and possession of household articles (a total of 14 items). Physical activity was assessed in the past month for occupational, recreational, commuting and other common daily activities [20]. The frequency and average duration was collected for each activity to calculate metabolic equivalent tasks (METs), where 1 MET is equivalent to expending 1 kcal/kg/h, which corresponds to the resting metabolic rate of sitting quietly [20].

### Dietary assessment

Diet was assessed using a validated interviewer-administered semi-quantitative food frequency questionnaire (FFQ) [21]. The FFQ collected information on portion size and frequency of 184 commonly consumed food items over the last 1 year. Standard portion size (e.g., tablespoon, ladle, and bowl) and frequency (daily, weekly, monthly, yearly/never) were recorded with the use of visual aids. A single FFQ was designed to cover dietary patterns across the four main regions of the study. Recipes were collected to generate databases of the food group composition of each food item across the four regions, and to calculate average daily food group intake. Nutrient databases were used to calculate the macro and micro-nutrient content of each recipe using Indian food composition tables [22] and the United States Department of Agriculture nutrient database (USDA, Release No. 14) [23] or McCance and Widdowson’s Composition of Foods, [24] were used where nutrient values were unavailable from the Indian food composition tables. Total energy, protein, fat, fibre, iron, calcium, zinc, folate, vitamin C and B12 intakes were calculated. A sub-sample was re-interviewed after completion of the FFQ (1–2 months, *n* = 185 and 12 months later, *n* = 305), yielding kappa coefficients = 0.26–0.71, [21] which are similar to reliability estimates from other studies [25, 26]. Three 24-h recalls were implemented in a sub-sample of participants (*n* = 530, 53.9% male) to validate the FFQ. The energy adjusted spearman correlation coefficients for macro-nutrients ranged from 0.43 (fibre) to 0.52 (fats) based on comparisons of FFQ with 24-h recalls [21]. For the present study FFQ responses were complete for 7067 participants, 182 food items (2 food items, *bhagar* and *kesari bhath*, from the grains and sweets categories respectively, were removed due to a high number of missing values) were classified into 30 food groups on the basis of nutrient and culinary similarities (some individual foods such as plain rice, tea and coffee were retained as separate food groups), for identification of dietary patterns. The details have been published elsewhere [9].

### Body mass index (BMI)

A digital personal scale (Beurer Model PS16, Ulm, Germany) accurate to 0.1 kg and stadiometer accurate to 1 mm (Leicester height measure (Chasmors Ltd. London UK) were used by trained personnel to record weight and height respectively of the participants in light indoor clothes without shoes [27].

### Blood pressure

Blood pressure was measured on the right upper arm with the participant in the sitting position after a rest of 5 min. Two readings were taken using an appropriate-sized cuff connected to a digital device (model M5-I; Omron-Matsusaka Company, Matsusaka City, Japan). Hypertension included doctor-diagnosed disease and/or a systolic BP ≥ 140 mmHg or a diastolic BP ≥ 90 mmHg at the time of the interview. Diabetes included doctor-diagnosed disease and/or a fasting plasma glucose criterion of 7.0 mmol/l.

### Biological outcome variables’ assessment

Ten ml of fasting (> 8 h) blood sample was collected by trained phlebotomists at the field sites, centrifuged at 2500 rpm for 15 min to separate serum or plasma. After processing (within an hour of collection), samples were stored at -20 °C in deep freezers for two to 3 weeks at field sites, after which they were transported to the laboratory at the All India Institute of Medical Sciences (AIIMS), New Delhi where they were stored at -80 °C ultra low deep freezer for bio-chemical analyses. Serum high density lipoprotein cholesterol level was measured directly using the elimination method, total cholesterol level was measured using an enzymatic endpoint method, triglyceride level was measured using GPO-PAP method, and glucose was measured using the GOD-PAP method using kits from Randox Laboratory Ltd. (Crumlin City, United Kingdom). LDL-C was calculated using Friedewald formula [28]. LDL-C was calculated for participants with TG levels < 400 mg/dl. For participants with TG levels≥400 mg/dL (*N* = 50) LDL-C levels were treated as missing. As part of local internal quality control, duplicate assays were performed and evaluated by AIIMS. The cardiac-biochemistry laboratory was part of external quality assurance program (RIQAS) from Randox.

### Statistical analysis

Principal components analysis (PCA) was used to identify major dietary patterns (DPs). The details of the statistical methods have been published in our earlier work [9]. Principal components generated were rotated by an orthogonal rotation (varimax) to increase interpretability. An eigenvalue cut-off > 1, scree plot and component interpretability were used to decide the number of components to retain, which were labelled on the basis of meaningful interpretation of component loadings and previous literature. Component scores were then generated for each retained component for use in the final analysis, as follows: the intake of each food group (per day average consumption in g) was weighted by its appropriate component loading on a principal component, and all weighted intakes were then summed to obtain a component score for that principal component, for each individual. Thus, we identified three major dietary patterns in this population: ‘cereal-savoury’ ‘fruit-vegetable-sweets-snacks’ and ‘animal food’ patterns. Final analysis for lipid outcomes was based on 7067 participants aged 18 years and above. For further analysis on fasting blood glucose outcome, known diabetics (*n* = 486, 6.9%) were excluded as diagnosis with diabetes may change glucose levels and also dietary habits. Thus, this analysis was based on 6581 participants aged 18 years and above.

One-way ANOVA and Chi-square tests were used for descriptive analysis. Robust standard error multivariable linear regression models adjusting for age, sex, standard of living index (SLI) (continuous), education (categories; no formal education, primary school, secondary school and beyond secondary school), geographical region (Lucknow, Nagpur, Hyderabad and Bangalore), migration status (urban, rural and urban migrant), BMI (continuous), tobacco use (categories), alcohol consumption (categories), energy intake (continuous), physical activity (total METS), regular use of any medication (yes/no) were used to investigate the association of different dietary patterns (using tertiles as cut-off) with total cholesterol (TC), low density lipoprotein-cholesterol (LDL-C), high density lipoprotein-cholesterol (HDL-C), triglycerides (TG), fasting blood glucose (FBG) and blood pressure (systolic and diastolic) levels at the significance level of 5%. All statistical analyses were conducted using STATA software version 10 (Stata-Corp.2009.Stata Statistical Software: Release 10. StataCorp LP).

## Results

### Characteristics of the dietary patterns

Additional file 1: Table S1 shows the component loadings of food groups on the three major dietary patterns (DPs) identified. There were 7 components which met the eigenvalue > 1 criterion; of these only the first 3 were considered for further analysis on the basis of the scree plot and their interpretability. When we visually examined the scree plot, we observed a sharp drop after the first three components, after which the curve became substantially shallower (data not shown). In addition, when we examined the contributions of individual food groups to the various components, the interpretability of the last four components in terms of what kind of composite dietary patterns they represented was unclear; i.e. they didn’t seem to represent cohesive dietary patterns with clear interpretability. Together, the first 3 components explained 28.6% of the total variance.

Component 1 loaded positively on whole/refined grains cooked with other food items, plain rice, rice-based dishes, coffee, nuts, snacks, condiments and soups, and negatively on potato, other vegetables (i.e., other than green leafy vegetables), and cooked milk products. Due to its positive loading on three cereal-groups, and on savoury foods such as nuts, snacks, and condiments, this component was termed the ‘cereal-savoury foods’ dietary pattern (Additional file 1: Table S1a).

Component 2 loaded positively on western cereals (e.g., corn/cereal flakes, bread/toast/rolls/buns, pizza/burger and noodles/macaroni/pasta), other vegetables (e.g., carrots, beet root, gourds, capsicum etc.), fruit, fruit juices, cooked milk products, snacks, condiments, sugars, sweets, and negatively on coffee. As this component loaded positively on fruits, snacks and sweets, and was the only component of the three to load on vegetables, it was termed the ‘fruit-veg- sweets-snacks’ dietary pattern (Additional file 1: Table S1a).

Component 3 loaded positively on all animal-food groups (red meat, poultry, fish/seafood, eggs, and other foods made with mutton/chicken) except other non-vegetarian food (organ meats, rabbit), the dairy-based categories and fats; hence it was termed the ‘animal food’ dietary pattern (Additional file 1: Table S1a).

### Sample characteristics

Sample characteristics by geographical region are summarized in Table 1. The mean age of participants was 40.8 (±10.3) years, with 41.6% female, 32.3% urban residents, 9% being illiterate, and 10.4%, 14.0% and 16.1% who were current tobacco smokers, tobacco chewers and alcohol drinkers respectively. Nagpur had the highest proportion of tobacco chewers (28.9%) while Hyderabad had the highest proportion of smokers (12.8%) and alcohol users (31.6%). Across the four regions, the prevalence of hypertension ranged from 5.9 to 33.4% and the prevalence of diabetes ranged from 1.6 to 13.3%. Overall, the average BMI was 23.8 (±4.5) kg/m2 and the average amount of physical activity expressed in total Metabolic Equivalent Tasks (METS) was 38.8(±4.6) hr./week. Sample characteristics according to dietary patterns (lowest and highest tertiles) are summarized in Additional file 1: Table S1b. The ‘cereal-savoury’ and ‘animal food’ patterns were consumed more in the Southern regions of Hyderabad and Bangalore and the ‘fruit-vegetable-sweets-snacks’ pattern was largely consumed in Lucknow (North) and Nagpur (Central).

**Table 1:** Socio-demographic and lifestyle characteristics^*^ of the Indian Migration Study participants by different geographical regions (*N* = 7067)

%/ mean(SD)	Lucknow *N* = 2000	Nagpur *N* = 1640	Hyderabad *N* = 1995	Bangalore *N* = 1432	Total *N* = 7067
Age (years)	43.8(8.5)	29.5(6.4)	44.7(9.1)	43.9(8.6)	40.8(10.3)
Gender					
Male	61.5	62.9	52.8	56.3	58.3
Female	38.5	37.0	47.2	43.7	41.6
Illiterate	7.0	2.0	19.7	5.2	9.0
Standard of living index^a^	22.7(6.4)	17.8(5.2)	19.2(6.9)	20.0(5.8)	20.0(6.4)
Migrant Status					
Rural	28.6	28.9	33.6	27.3	29.8
Migrants	28.6	29.0	33.6	27.4	29.8
Urban	41.0	28.9	19.2	43.1	32.3
Smoking					
Never	86.7	92.0	84.3	86.3	87.2
Former	1.8	1.5	2.8	3.2	2.3
Current	11.4	6.4	12.8	10.4	10.4
Tobacco Chewing					
Never	80.7	69.3	90.5	95.2	83.8
Former	2.6	1.7	1.9	2.2	2.1
Current	16.6	28.9	7.5	2.5	14.0
Alcohol					
Never	86.5	88.5	64.8	87.1	80.7
Former	2.6	2.3	4.4	2.7	3.1
Current	10.8	9.2	31.6	10.1	16.1
Diabetes ^b^					
No	86.7	97.4	88.7	86.8	89.8
Yes	13.3	1.6	11.1	12.5	9.8
Hypertension^b^					
No	70.6	93.8	66.5	72.8	75.2
Yes	29.4	5.9	33.4	27.0	24.6
Systolic blood pressure mm/Hg	123.4 (17.2)	113.8 (11.7)	127.3 (18.9)	123.1 (17.3)	122.2 (17.4)
Diastolic blood pressure mm/Hg	77.3 (10.7)	73.0 (8.7)	81.6 (11.3)	79.2 (10.9)	77.9 (10.9)
Physical activity METS^c^ hr./week	38.7(4.0)	40.2(5.0)	37.9(4.7)	38.7(4.3)	38.8(4.6)
BMI^d^ kg/m^2^	24.4(4.4)	20.9(3.3)	24.6(4.4)	25.0(4.3)	23.8(4.5)
Dietary patterns in tertiles					
Cereal-savoury foods pattern					
T1	89.6	33.1	0.9	0.07	33.3
T2	9.4	57.9	49.1	16.6	33.3
T3	0.9	8.9	49.9	83.3	33.3
Fruits-vegetables-sweets-snacks pattern					
T1	20.3	17.2	53.1	42.2	33.3
T2	41.2	34.3	26.3	30.9	33.3
T3	38.4	48.3	20.5	26.8	33.3
Animal food pattern (fish-red meat-egg-poultry)					
T1	51.4	39.7	15.4	25.7	33.3
T2	34.9	33.0	34.8	29.4	33.3
T3	13.7	27.2	49.7	44.9	33.3
Biomarker levels [mean (±SD)]					
Fasting blood glucose mmol/l	5.7 (1.8)	4.8 (0.9)	5.1 (0.8)	5.5 (1.7)	5.3 (1.4)
Total cholesterol mmol/l	4.7 (1.1)	4.4 (1.1)	4.8 (1.1)	4.9 (1.1)	4.7 (1.1)
Low-density lipoprotein cholesterol mmol/l	2.8 (0.9)	2.7 (0.9)	2.9 (1.0)	3.1 (0.9)	2.9 (0.9)
High-density lipoprotein cholesterol mmol/l	1.2 (0.2)	1.1 (0.2)	1.1 (0.2)	1.1 (0.2)	1.2 (0.2)
Triglycerides mmol/l	1.5 (0.8)	1.3 (0.6)	1.5 (0.8)	1.5 (0.9)	1.5 (0.8)

^*^p-values for difference in means or proportions are from ANOVA for continuous data and Chi-square test of significance for categorical data (we found a *p* < 0.0001 for all comparisons between the regions)

^a^Standard of Living Index (SLI) distribution is 1–36 (Median 23, IQR =17–27)

^b^Hypertension included doctor-diagnosed disease and/or a systolic BP ≥ 140 mmHg or a diastolic BP ≥ 90 mmHg at the time of the interview. Diabetes included doctor-diagnosed disease and/or a fasting plasma glucose criterion of > 7.0 mmol/l [33]

^c^*METS* Metabolic Equivalent Tasks

^d^*BMI* Body Mass Index

### Association of dietary patterns with blood lipids, fasting glucose and blood pressure

Multivariable adjusted linear association of major dietary patterns with blood lipids, fasting glucose and systolic and diastolic blood pressure using robust standard error are presented in Table 2. The ‘cereal-savoury foods’ and ‘fruits-vegetables-sweets-snacks’ patterns did not show any association with blood lipids and fasting glucose levels. Higher intake of ‘fruits-vegetables-sweets-snacks’ pattern was positively associated with diastolic blood pressure (β coefficient T3 vs T1 1.2 mm/Hg; 95% CI: 0.8, 1.2 mm/Hg; p-trend = 0.014). Higher intake of ‘animal food’ pattern (‘fish-red meat-egg-poultry’) was positively associated with total cholesterol, LDL-C, HDL-C, fasting blood glucose (FBG) as well as with systolic and diastolic blood pressure (SBP and DBP) levels (β coefficient T3 vs T1 - TC: 0.10 mmol/L; 95% CI: 0.02, 0.17 mmol/L; p-trend = 0.013; LDL-C: 0.07 mmol/L; 95% CI: 0.004, 0.14 mmol/L; p-trend = 0.041; HDL-C: 0.02 mmol/L; 95% CI: 0.004, 0.04 mmol/L; p-trend = 0.016; FBG: 0.09 mmol/L; 95% CI: 0.01, 0.16 mmol/L; p-trend = 0.021; SBP: 1.2 mm/Hg; 95% CI: 0.1, 2.3 mm/Hg; p-trend = 0.032; DBP: 0.9 mm/Hg; 95% CI: 0.2, 1.5 mm/Hg; p-trend = 0.013) (Table 2).

**Table 2:** Multivariable-adjusted linear associations^a^ (beta co-efficient, 95% confidence interval) of major dietary patterns with cardio-metabolic risk factors in the Indian Migration Study participants (*N* = 7067)

Food patterns Tertiles	Total cholesterol mmol/L		LDL-C mmol/L		Triglycerides mmol/L		HDL-C mmol/L		Fasting glucose^b^ mmol/L		Systolic blood pressure mm/Hg		Diastolic blood pressure mm/Hg	
	Age, sex adjusted	Fully adjusted^a^	Age, sex adjusted	Fully adjusted^a^	Age, sex adjusted	Fully adjusted^a^	Age, sex adjusted	Fully adjusted^a^	Age, sex adjusted	Fully adjusted^a^	Age, sex adjusted	Fully adjusted^a^	Age, sex adjusted	Fully adjusted^a^
Cereal savoury food pattern														
TI	Ref	Ref	Ref	Ref	Ref	Ref	Ref	Ref	Ref	Ref	Ref	Ref	Ref	Ref
T2	−0.03 (−0.09, 0.03)	−0.05 (− 0.15, 0.04)	0.04 (− 0.01, 0.10)	− 0.03 (− 0.12, 0.05)	0.01 (− 0.02, 0.05)	− 0.009 (− 0.06, 0.04)	− 0.07 (− 0.09, − 0.06)	− 0.01 (− 0.03, 0.01)	−0.37 (− 0.46, − 0.29)	0.007 (− 0.07, 0.08)	1.8 (0.8, 2.7)	0.1 (−1.1,1.3)	1.9 (1.2, 2.5)	− 0.1 (− 0.9, 0.7)
T3	0.12 (0.05, 0.19)	− 0.009 (− 0.13, 0.12)	0.19 (0.13, 0.25)	0.001 (− 0.11, 0.11)	0.09 (0.04, 0.14)	0.01 (− 0.06, 0.1)	−0.10 (− 0.12, − 0.09)	−0.02 (− 0.04, 0.007)	−0.30 (− 0.40,-0.21)	−0.01 (− 0.11, 0.08)	1.3 (0.3, 2.3)	−0.7 (−2.4, 0.9)	2.5 (1.9, 3.2)	− 0.9 (− 1.9, 0.2)
p-trend	0.001	0.946	< 0.0001	0.880	< 0.0001	0.540	< 0.0001	0.153	< 0.0001	0.745	Ref	Ref	Ref	Ref
Fruit-veg-sweet-snack pattern														
TI	Ref	Ref	Ref	Ref	Ref	Ref	Ref	Ref	Ref	Ref	Ref	Ref	Ref	Ref
T2	0.06 (−0.002, 0.13)	0.02 (−0.05, 0.1)	0.03 (− 0.03, 0.08)	0.01 (− 0.05, 0.08)	0.03 (− 0.01, 0.07)	0.02 (− 0.02, 0.07)	0.02 (0.008, 0.03)	−0.0001 (− 0.01, 0.01)	0.06 (− 0.01, 0.05)	−0.01 (− 0.08, 0.06)	−0.7 (−1.7, 2.0)	0.3 (− 0.8, 1.3)	−0.2 (− 0.8, 0.5)	−0.6 (− 0.06, 1.3)
T3	0.04 (− 0.02, 0.11)	−0.02 (− 0.11, 0.07)	0.01 (− 0.05, 0.07)	−0.01 (− 0.09, 0.06)	0.02 (− 0.02, 0.06)	−0.02 (− 0.08, 0.03)	0.01 (0.002, 0.03)	−0.005 (− 0.02, 0.01)	0.03 (− 0.11, 0.03)	−0.06 (− 0.14, 0.02)	−0.3(− 1.2, 0.7)	1.2 (− 0.1, 2.5)	−0.02 (− 0.6, 0.6)	1.2 (0.8, 1.2)
p-trend	0.209	0.604	0.715	0.661	0.341	0.472	0.025	0.624	0.331	0.143	0.555	0.067	0.953	0.014
Animal food pattern (Fish-red meat-egg-poultry)														
TI	Ref	Ref	Ref	Ref	Ref	Ref	Ref	Ref	Ref	Ref	Ref	Ref	Ref	Ref
T2	0.004 (−0.06, 0.06)	0.01 (−0.05, 0.08)	0.006 (−0.05, 0.06)	0.002 (− 0.05, 0 .06)	0.02 (− 0.01, 0.07)	0.02 (− 0.03, 0.06)	−0.01 (− 0.02, 0.003)	0.006 (− 0.008, 0.02)	−0.08 (− 0.17, − 0.001)	0.01 (− 0.05, 0.07)	1.4 (0.5,2.4)	0.5 (− 0.4, 1.5)	1.0 (0.4, 1.6)	0.2 (− 0.4, 0.8)
T3	0.17 (0.10, 0.24)	0.10 (0.02, 0.17)	0.15 (0.09, 0.1)	0.07 (0.004, 0.14)	0.11 (0.06, 0.16)	0.04 (−0.01, 0.09)	−0.02 (− 0.03, − 0.006)	0.02 (0.004, 0.04)	−0.09 (− 0.18, − 0.009)	0.09 (0.01, 0.16)	2.8 (1.8,3.8)	1.2 (0.1, 2.3)	2.9 (2.3, 3.6)	0.9 (0.2, 1.5)
p-trend	< 0.0001	0.013	< 0.0001	0.041	< 0.0001	0.181	0.005	0.016	0.029	0.021	< 0.001	0.032	< 0.001	0.013

^a^Robust standard error adjusted for age (continuous in years), sex (male/female), migration status (rural, urban, urban migrant), site (Lucknow, Nagpur, Hyderabad, Bangalore), SLI (continuous score), education (no formal education, primary school, secondary school and beyond secondary school), BMI (continuous in kg/m^2^), total energy (continuous in kcal/day), physical activity (continuous in totalMETS), tobacco (never, past, current), alcohol (never, past, current), use of any regular medication for chronic conditions such as diabetes, hypertension and/or food supplements (yes/no), sib-pair

^b^Analysis excluded known diabetics (*n* = 486)

### Sensitivity analyses

‘Fruits-vegetables-sweets-snacks’ pattern was found to be highly variable across different regions of India in terms of component loadings for individual food components (based on our earlier work [9] in the same study population); and there were fewer number of participants in certain categories of the cereal-savoury pattern (*N* = 19 in the highest tertile in Lucknow; *N* = 19 in Hyderabad and *N* = 1 in Bangalore in the lowest tertile) making any sensitivity analysis less meaningful for these patterns. In addition, these dietary patterns showed overall no associations with risk parameters except for a solitary positive association between intake of the ‘fruits-vegetables-sweets-snacks’ pattern and diastolic blood pressure. Thus, we restricted this analysis to ‘animal food’ pattern which was the most consistent pattern in terms of component loadings across all regions of India [9].

We conducted sensitivity analyses to evaluate if there were differences in associations of the ‘animal food’ pattern with cardio-metabolic risk factors based on regions (Lucknow, Nagpur, Bangalore and Hyderabad), locations (urban/ urban migrant/ rural) and standard of living index (SLI; low, medium and high). We also did sensitivity analyses examining the associations of daily consumption of individual animal foods (fish, red meat, egg and poultry in tertiles) with blood lipids, fasting blood glucose and blood pressure, further adjusting for the cereal-savoury foods and fruits-vegetables-sweets-snacks patterns as an attempt to increase the understanding of individual components of ‘animal food’ dietary pattern in India. Although attenuated, the associations were in similar direction, across regions (Additional file 1: Table S2a), locations (Additional file 1: Table S2b) and SLI (Additional file 1: Table S2c) with few interactions between the ‘animal food’ pattern and regions, locations or SLI. Nevertheless, there were significant interactions between the ‘animal food’ dietary pattern and regions for associations with triglycerides levels (Additional file 1: Table S2a) and the ‘animal food’ dietary pattern and SLI for associations with fasting blood glucose levels (Additional file 1: Table S2c). High intake of individual ‘animal food’ components (fish, red meat, poultry and eggs in tertiles) also showed positive associations with the cardio-metabolic risk factors (Additional file 1: Table S3).

## Discussion

Across four geographical regions spanning 18 states of India, three major dietary patterns - ‘cereals-savoury foods’, ‘fruit-veg-sweets-snacks’ and ‘animal food’, were identified through principal components analysis (PCA) of dietary intake data in a large cross-sectional study of rural and urban populations. High intake of the ‘animal food’ pattern was associated with high levels of fasting blood glucose, and total, LDL, and HDL cholesterol as well as systolic and diastolic blood pressure after adjusting for several potential confounders and was consistent in sensitivity analyses. No association was seen for the other largely plant-based dietary patterns except for a solitary positive association with diastolic blood pressure for high intake of ‘fruits-vegetables-sweets-snacks’ pattern. In a previous analysis with the same IMS data, we found positive associations of the ‘animal food’ pattern with obesity and abdominal adiposity, in line with our current study [9].

While ‘Prudent’ (high intake of vegetables, fruit, legumes, whole grains, and seafood), ‘Mediterranean’ (higher intake of vegetables, legumes, fruits, nuts, whole grains, cheese or yogurt, fish, and monounsaturated relative to saturated fatty acids), and ‘DASH’ (fruit, vegetables and low fat dairy products) dietary patterns have been found to be inversely associated with cardiovascular diseases and type-II diabetes, the ‘Western’ dietary pattern characterized by a high intake of processed and red meat, butter and other high-fat dairy products, eggs, and refined grains, has been associated with an increased risk of CVD and type-II diabetes [4, 7, 8].

The three dietary patterns identified here were distinct for our study population and were a blend of healthy and unhealthy aspects of the above mentioned dietary patterns. For example, ‘cereals-savoury foods’ showed positive loadings for nuts and whole grains but also had positive loadings for refined grains and negative loadings for vegetables; ‘fruit-veg-sweets-snacks’ pattern showed positive loadings for fruits and vegetables as well as for snacks and sugar. This could have partly attributed to the null associations in general with cardio-metabolic risk factors for these patterns. The positive association of diastolic blood pressure with high intake of ‘fruits-vegetables-sweets-snacks’ pattern could possibly be attributed to snacks which tend to have a high salt content.

The ‘animal food’ pattern in our study, the only consistent dietary pattern across all regions and locations of our study population, was a blend of components of prudent (fish) and western (red meat, eggs) dietary patterns and was positively associated with cardio-metabolic risk factors. This was further confirmed in sensitivity analyses where we stratified by regions, locations and SLI. We also found positive associations with cardio-metabolic risk factors for high intake of individual components such as red meat, eggs, poultry and fish. Several potential mechanisms through which the ‘animal food’ patterns could have influenced cardio-metabolic risk factors in this population can be proposed, although further studies are required to confirm the findings. For example, imbalance in saturated and unsaturated fat (high saturated fat and low unsaturated fat) and potentially low fibre in the ‘animal food’ coupled with high salt content in cooked animal food could have influenced blood lipids and blood pressure levels respectively [29]. The positive association of high intake of ‘fruits-vegetables-sweets-snacks’ with diastolic blood pressure lends some support to this theory as high salt content in snacks could have driven the observed association. A study based on data from India’s third National Family Health Survey (NFHS-3) that showed a positive association of fish consumption with diabetes (OR: 2.02; 95% CI, 1.59–2.57) alluded that the pattern of cooking such as deep frying with high amounts of cooking oils could have contributed to the positive findings [30]. A study conducted among South Asians (84% Indian origin) living in the US found high total cholesterol (β: 0.45 mmol/L (95% CI: 0.11 to 0.78 mmol/L)) and LDL- C (β: 0.31 mmol/L (95% CI: 0.02 to 0.61 mmol/L)) with high intake of animal protein pattern [31] similar to our study. They found no association of lipids and glucose levels with any other patterns such as ‘fried snacks, sweets, and high-fat dairy’ and ‘fruits, vegetables, nuts, and legumes’ except for an inverse association with HDL-C for high intake of ‘fried snacks, sweets, and high-fat dairy’ pattern (*p* < 0.0001), although ‘fruits, vegetables, nuts, and legumes’ pattern was inversely associated with the odds of metabolic syndrome and hypertension and ‘fried snacks, sweets, and high-fat dairy’ pattern was directly associated with insulin resistance [31].

Indian studies evaluating dietary patterns as they relate with CVD and diabetes risk factors are limited [9, 10, 13, 32, 33]. A study among women in West Bengal found three dietary patterns including ‘vegetables fruit and pulses’ pattern which was similar to our ‘fruit-veg-sweets-snacks’ pattern but also had positive loadings for poultry and eggs [32]. This pattern had an inverse association with total cholesterol and LDL-C. In the same study the ‘hydrogenated and saturated fat and vegetable oil’ pattern was positively associated with BMI, waist circumference and HDL-C but no association was found for red meat and high-fat dairy pattern (which also positively loaded for whole and refined grains and low-fat dairy) with any risk factors [32]. Another study in Delhi, Trivandrum and Mumbai, also found specific regional patterns, such as ‘fruit-dairy’ and ‘vegetables-pulses’ in New Delhi, ‘pulses-rice’ and ‘sweets-snacks’ in Trivandrum and ‘fruits-vegetables’ and ‘snacks-meat’ in Mumbai, though the authors did not evaluate associations with lipid and glucose levels [13]. Previously, IMS investigators found five clusters of people (rice and low diversity; rice and fruit; wheat and pulses; wheat, rice and oils; rice and meat) [33] using latent class analysis (LCA) and these clusters were associated with cardio-metabolic risk factors. We are adding to that by examining the IMS dietary data in a different way, using PCA, which may be more applicable because this population is heterogeneous in terms of dietary consumption and it involved participants from over 18 different regions of India consisting of urban, rural as well as migrant population. We have found differences in food consumption patterns in this population based on regions and locations [34].

Through one of our earlier analyses in this populations based on simple lacto-vegetarian and non-vegetarian patterns we found beneficial associations of vegetarian diet with CVD and diabetes risk factors including blood lipids, fasting glucose and blood pressure [29]. At the same time we also found reduced bio-availability of vitamin B12 with Indian vegetarian diets [35] which could be a predictor for various chronic diseases particularly CVD [36]. A more detailed analysis of dietary patterns is needed to facilitate the evaluation of aggregate effects of different food components and nutrients consumed in the Indian context [9].

In India, although there are important regional differences in DPs [13], the ‘animal food’ pattern showed considerable consistency across regions, urban-rural locations and standard of living. The quantity and pattern of ‘animal food’ consumption in India are different from their Western counterparts. For example while the median intake is about 49–65 g/day for meat and 33–34 g/day for fish in the West [37–39], the consumption is only about 20.3(9.6–38.9) g/day for meat and 3.9 (0.5–9.9) g/day for fish in our population. Similarly, the Western ‘animal foods’ dietary pattern largely includes processed meat, red meat and eggs with high fat foods and refined grains, while in our study the ‘animal foods’ pattern was consumption of red meat, fish, eggs and poultry [37–39]. Despite these differences, we found positive associations of the ‘animal food pattern with cardio-metabolic risk factors in India, potentially implying the role of differing cooking patterns [30], in addition to the components of animal food (e.g., red meat). As quantity of everyday consumption of ‘animal food’ in India is very less compared to Western counterparts, cooking pattern is a factor that holds clinical and public health relevance, if proven by further studies, for potential interventions.

In this study, we used a validated 184-item semi-quantitative food frequency questionnaire (FFQ) to capture the wide range of diets consumed across four different regions of India, and obtained fasting blood samples for measurement of biochemical markers. We used data reduction techniques to identify composite dietary patterns in India. The cross-sectional study design and possible measurement error in dietary intake are the major limitations of the study with potential impact on the study results, which should be interpreted with caution. However, the large, diverse and heterogeneous nature of this study population and their diets and consistency of associations across several sensitivity analyses for ‘animal food’ pattern, are strengths of the study. Future nutrition research in India should replicate these findings in prospective studies and evaluate specific public health dietary interventions (e.g., lower consumption of certain animal food components, modification of cooking patterns etc.).

## Conclusion

High intake of the ‘animal food’ dietary pattern was positively associated with cardio-metabolic risk factors in India. Our findings possibly indicate that cooking pattern is partly driving the association. Given the cross-sectional nature of this study, comprehensive longitudinal evaluations of diet are needed to better understand and validate the findings to devise potential means of interventions for combatting NCDs in India which could have large public health impact in this population.

## Additional file

Additional file 1: **Table S1 (a):** Component loadings for the 3 main dietary patterns (DPs) identified using principal components analysis (PCA) of FFQ data from the Indian Migration Study*. **Table S1(b):** Socio-demographic and lifestyle characteristics of the Indian Migration study participants in the first (T1) and third (T3) tertiles of the three dietary patterns (N=7067). **Table S2 (a):** Multivariable-adjusted linear associations* (beta co-efficient, 95% confidence interval) of ‘animal food’ pattern with cardio-metabolic risk factors by four different regions of the Indian Migration Study. **Table S2 (b):** Multivariable-adjusted linear associations* (beta co-efficient, 95% confidence interval) of ‘animal food’ pattern with cardio-metabolic risk factors by different locations of the Indian Migration Study. **Table S2 (c):** Multivariable-adjusted linear associations* (beta co-efficient, 95% confidence interval) of ‘animal food’ pattern with cardio-metabolic risk factors by different standard of living (SLI) of the Indian Migration Study.**Table S3:** Multivariable adjusted* associations (beta co-efficient, 95% confidence interval) of daily consumption of individual animal food components in tertiles (fish /red meat/poultry/eggs) with cardio-metabolic risk factors of the Indian Migration Study participants (N=7067). (DOCX 70 kb)

## Abbreviations

BMI: Body mass index
CVD: Cardiovascular disease
DALY: Disability adjusted life years
DASH: Dietary approaches to stop hypertension
DP: Dietary pattern
FFQ: Food frequency questionnaire
HDL-C: High density lipoprotein-cholesterol
IMS: Indian migration study
LDL-C: Low density lipoprotein-cholesterol
LMIC: Low middle income countries
METs: Metabolic equivalent tasks
NCD: Noncommunicable disease
PCA: Principal component analysis
SLI: Standard of living Index
TC: Total cholesterol
TG: Triglycerides and FBG-fasting blood glucose
USDA: United States Department of Agriculture
