# Supplementary material for: Association between empirically derived dietary patterns with blood lipids, fasting blood glucose and blood pressure in adults - the India migration study
Source: Nutr J. 2018 Feb 8;17:15. doi: 10.1186/s12937-018-0327-0 (PMC5806276; doi:10.1186/s12937-018-0327-0)
Supplement: Supplementary file 1 — Table S1 (a): Component loadings for the 3 main dietary patterns (DPs) identified using principal components analysis (PCA) of FFQ data from the Indian Migration Study*. Table S1(b): Socio-demographic and lifestyle characteristics of the Indian Migration study participants in the first (T1) and third (T3) tertiles of the three dietary patterns (N=7067). Table S2 (a): Multivariable-adjusted linear associations* (beta co-efficient, 95% confidence interval) of ‘animal food’ pattern with cardio-metabolic risk factors by four different regions of the Indian Migration Study. Table S2 (b): Multivariable-adjusted linear associations* (beta co-efficient, 95% confidence interval) of ‘animal food’ pattern with cardio-metabolic risk factors by different locations of the Indian Migration Study. Table S2 (c): Multivariable-adjusted linear associations* (beta co-efficient, 95% confidence interval) of ‘animal food’ pattern with cardio-metabolic risk factors by different standard of living (SLI) of the Indian Migration Study.Table S3: Multivariable adjusted* associations (beta co-efficient, 95% confidence interval) of daily consumption of individual animal food components in tertiles (fish /red meat/poultry/eggs) with cardio-metabolic risk factors of the Indian Migration Study participants (N=7067). (DOCX 70 kb) [file 12937_2018_327_MOESM1_ESM.docx]

**Table S1 (a):** Component loadings for the 3 main dietary patterns (DPs) identified using principal components analysis (PCA) of FFQ data from the Indian Migration Study *

| **Food group** | DP1  Cereal-savoury food pattern | DP2  Fruits-vegetables-sweets-snacks pattern | DP3  Animal food pattern  (Fish-red meat-egg-poultry pattern) |
| --- | --- | --- | --- |
| Whole grains |  |  |  |
| Whole/refined grains cooked† | 0.17 |  |  |
| Plain rice | 0.32 |  |  |
| Rice cooked† | 0.33 |  |  |
| Western cereals |  | 0.24 |  |
| Pulses & Legumes |  |  |  |
| Green leafy vegetables |  |  |  |
| Potato | -0.40 |  |  |
| Other vegetables | -0.31 | 0.21 |  |
| Fruits |  | 0.49 |  |
| Fruit juices |  | 0.27 |  |
| Milk & milk products |  |  |  |
| Milk & milk products cooked† | -0.16 | 0.22 |  |
| Red meat |  |  | 0.49 |
| Poultry |  |  | 0.47 |
| Fish and other sea food |  |  | 0.48 |
| Eggs |  |  | 0.32 |
| Other non-vegetarian |  |  |  |
| Mutton or chicken |  |  | 0.39 |
| Fats |  |  |  |
| Sugar |  | 0.26 |  |
| Alcohol |  |  |  |
| Tea |  |  |  |
| Coffee | 0.34 | -0.18 |  |
| Sugar sweetened beverages |  |  |  |
| Nuts | 0.17 |  |  |
| Snacks | 0.17 | 0.41 |  |
| Sweets and deserts |  | 0.41 |  |
| Condiments, pickles, chutneys | 0.35 | 0.24 |  |
| Soups | 0.31 |  |  |

Component loadings represent the magnitude and direction of the association of foods or food groups with dietary patterns (factors); Rotated factor pattern; values presented are correlation coefficients

*Absolute values <0.15 excluded from the table for simplicity

† With oil and/or other food items

**Table S1b:** Socio-demographic and lifestyle characteristics of the Indian Migration study participants in the first (T1) and third (T3) tertiles of the three dietary patterns (N=7067)

|  | **Cereal-savoury pattern** | | **Fruit-veg-sweet-snacks pattern** | | **Animal food pattern** | |
| --- | --- | --- | --- | --- | --- | --- |
| **% / mean (±SD)** | **T1** | **T3** | **T1** | **T3** | **T1** | **T3** |
| **Age** (years) | 40.9 (9.9) | 42.9 (9.3)* | 43.4 (10.2) | 38.4 (10.1)* | 40.5 (10.5) | 40.9 (9.9) |
| **Gender**  Male  Female | 63.1  36.9 | 58.0 *  42.0 | 53.6  46.3 | 64.4*  35.6 | 50.2  49.7 | 66.2*  33.8 |
| **Illiterate** | 6.3 | 7.3 | 17.9 | 3.5* | 6.3 | 8.4* |
| **Standard of Living Index †** | 21.5 (6.5) | 20.4 (5.9)* | 18.0 (7.0) | 21.2 (5.9)* | 20.9 (6.4) | 20.2 (6.0)* |
| **Migrant Status**  Rural  Migrants  Urban | 30.8  29.9  39.3 | 29.8**  33.5  36.7 | 42.2  30.5  27.3 | 27.3*  31.8  40.9 | 30.1  32.7  37.2 | 31.0  33.4  35.6 |
| **Smoking**  Never  Former  Current | 87.7  1.4  10.9 | 86.0*  2.9  11.0 | 85.1  2.5  12.5 | 87.8*  2.4  9.8 | 97.8  1.9  6.3 | 82.1*  3.1  14.9 |
| **Tobacco chewing**  Never  Former  Current | 77.7  2.2  20.0 | 91.8*  1.9  6.3 | 88.1  1.9  9.9 | 80.6*  2.2  17.2 | 87.3  1.9  10.7 | 82.9*  1.9  15.1 |
| **Alcohol**  Never  Former  Current | 86.6  2.5  10.9 | 76.9*  3.4  19.7 | 76.4  3.3  20.3 | 83.8*  2.9  13.2 | 93.4  1.9  4.7 | 68.9*  4.1  27.0 |
| **Diabetes *****  No  Yes | 89.5  10.5 | 88.1  11.9 | 87.0  12.9 | 92.7*  7.3 | 90.5  9.5 | 89.3  10.7 |
| **Hypertension*****  No  Yes | 75.9  24.1 | 72.9**  27.0 | 71.4  28.6 | 78.3*  21.7 | 77.5  22.5 | 73.6*  26.4 |
| Systolic blood pressure mm/Hg | 121.6 (16.6) | 123.9 (17.2)* | 124.1 (19.0) | 120.9 (15.7)* | 120.3 (16.7) | 124.0 (17.5)* |
| Diastolic blood pressure mm/Hg | 76.5 (10.4) | 79.7 (11.1)* | 78.8 (11.5) | 77.2 (10.3)* | 76.4 (10.4) | 79.6 (11.2)* |
| Physical activity METS ‡ hr/week | 39.1 (4.2) | 38.4 (4.5)* | 38.5 (4.8) | 39.4 (4.7)* | 38.8 (4.4) | 38.7 (4.7) |
| BMI ^ρ^ kg/m^2^ | 23.7 (4.4) | 24.8 (4.4)* | 23.8 (4.7) | 23.6 (4.3) | 23.6 (4.5) | 24.3 (4.2)* |
| **Biomarkers** | | | | | |  |
| Fasting blood glucose mmol/l | 5.5 (1.7) | 5.3 (1.3)* | 5.4 (1.5) | 5.2 (1.2)* | 5.3 (1.5) | 5.3 (1.3) |
| Total cholesterol mmol/l | 4.7 (1.1) | 4.8 (1.1)* | 4.7 (1.1) | 4.6 (1.1)** | 4.6 (1.1) | 4.8 (1.1)* |
| Low-density lipoprotein cholesterol mmol/l | 2.8 (0.9) | 3.0 (0.9)* | 2.9 (1.0) | 2.8 (0.9)** | 2.8 (0.9) | 3.0 (1.0)* |
| High-density lipoprotein cholesterol mmol/l | 1.2 (0.2) | 1.1 (0.2)* | 1.1 (0.2) | 1.2 (0.2) | 1.2 (0.2) | 1.1 (0.2)* |
| Triglycerides mmol/l | 1.4 (0.7) | 1.5 (0.8)* | 1.5 (0.8) | 1.4 (0.8) | 1.4 (0.8) | 1.5 (0.8)* |

p-values for difference in means or proportions are from ANOVA for continuous data and Chi-square test of significance for categorical data; *p<0.01 **p<0.05

† Standard of Living Index (SLI) distribution is 1-36 (Median 23, IQR =17-27)

‡ METS- Metabolic Equivalent Tasks

^ρ^ BMI – Body mass index

***Hypertension included doctor-diagnosed disease and/or a systolic BP ≥140 mm Hg or a diastolic BP ≥90 mm Hg at the time of the interview. Diabetes included doctor-diagnosed disease and/or a fasting plasma glucose criterion of >7.0 mmol/l [33].

**Table S2 (a):** Multivariable-adjusted linear associations* (beta co-efficient, 95% confidence interval) of ‘animal food’ pattern with cardio-metabolic risk factors by four different regions of the Indian Migration Study.

| Animal food pattern  tertiles | **Total cholesterol**  mmol/L | **LDL-C**  mmol/L | **Triglycerides**  mmol/L | **HDL-C**  mmol/L | **Fasting glucose**** mmol/L | **Systolic blood pressure** mm/Hg | **Diastolic blood pressure** mm/Hg |
| --- | --- | --- | --- | --- | --- | --- | --- |
| **Lucknow** | | | | | | | |
| TI  T2  T3 | Ref  -0.02(-0.13, 0.09)  0.08(-0.06,0.24) | Ref  -0.01(-0.11,0.09)  0.05 (-0.08,0.19) | Ref  -0.003(-0.07,0.06)  0.09(-0.03, 0.21) | Ref  -0.007(-0.03,0.01)  0.01(-0.02,0.04) | Ref  0.12(-0.007,0.25)  **0.22(0.05,0.39)** | Ref  -0.5 (-1.0, 2.1)  2.5 (-0.1, 5.1) | Ref  -0.4 (-0.6, 1.4)  1.9 (0.3, 3.5) |
| **p-trend** | 0.468 | 0.622 | 0.233 | 0.826 | 0.005 | 0.075 | 0.030 |
| **Nagpur** | | | | | | | |
| TI  T2  T3 | Ref  **0.19(0.03,0.34)**  0.15(-0.01,0.32) | Ref  0.12(-0.004,0.25)  0.11(-0.03,0.26) | Ref  **0.12(0.03,0.20)**  0.09(-0.0004,0.18) | Ref  0.02(-0.008,0.05)  0.02(-0.01,0.05) | Ref  -0.06(-0.16,0.04)  0.06(-0.06,0.18) | Ref  0.4 (-0.9, 1.8)  0.5 (-1.1, 2.1) | Ref  0.2 (-0.8, 1.2)  0.4 (-0.8, 1.5) |
| **p-trend** | 0.05 | 0.106 | 0.029 | 0.185 | 0.434 | 0.505 | 0.479 |
| **Hyderabad** | | | | | | | |
| TI  T2  T3 | Ref  0.04(-0.12,0.20)  0.11(-0.04,0.27) | Ref  0.04 (-0.10,0.18)  0.12(-0.01,0.26) | Ref  -0.007(-0.11,0.10)  -0.02(-0.14,0.08) | Ref  0.01(-0.02,0.04)  0.02(-0.01,0.05) | Ref  0.04(-0.03,0.11)  0.03(-0.07,0.12) | Ref  1.9 (-0.4, 4.4)  **3.4 (0.9, 5.8)** | Ref  0.7 (-0.8, 2.1)  1.7(0.2, 3.1) |
| **p-trend** | 0.123 | 0.068 | 0.568 | 0.222 | 0.717 | 0.007 | 0.015 |
| **Bangalore** | | | | | | | |
| TI  T2  T3 | Ref  **-0.15(-0.29, -0.001)**  0.07(-0.08,0.23) | Ref  **-0.14(-0.27, -0.008)**  0.02(-0.11,0.17) | Ref  -0.04(-0.16,0.07)  -0.04(-0.09,0.18) | Ref  -0.002(-0.03,0.03)  0.02(-0.007,0.06) | Ref  -0.15(-0.32,0.02)  0.04(-0.15,0.23) | Ref  -1.4 (-3.8, 0.9)  -1.3 (-3.6, 0.9) | Ref  -1.1(-2.6, 3.0)  -0.4 (-1.8, 0.9) |
| **p-trend** | 0.253 | 0.511 | 0.450 | 0.096 | 0.562 | 0.291 | 0.656 |
| p for interaction^#^ between animal food pattern and sites | 0.1090 | 0.2343 | **0.0373** | 0.4022 | 0.0967 | 0.5370 | 0.2541 |

*Robust standard error adjusted for age (continuous in years), sex (male/female), migration status (rural, urban, urban migrant), site(Lucknow, Nagpur, Hyderabad, Bangalore), SLI(continuous score), education (no formal education, primary school, secondary school and beyond secondary school), BMI(continuous in kg/m^2^), total energy(continuous in kcal/day), physical activity(continuous in totalMETS), tobacco(never, past, current), alcohol (never, past, current), use of any regular medication for chronic conditions such as diabetes, hypertension and/or food supplements (yes/no), sib-pair.

. **Analysis excluded known diabetics (n=486). #Wald test for interaction; Significant associations in bold

**Table S2 (b):** Multivariable-adjusted linear associations* (beta co-efficient, 95% confidence interval) of ‘animal food’ pattern with cardio-metabolic risk factors by different locations of the Indian Migration Study.

| Animal food pattern  tertiles | **Total cholesterol**  mmol/L | **LDL-C**  mmol/L | **Triglycerides**  mmol/L | **HDL-C**  mmol/L | **Fasting glucose****  mmol/L | **Systolic blood pressure** mm/Hg | **Diastolic blood pressure** mm/Hg |
| --- | --- | --- | --- | --- | --- | --- | --- |
| **Rural** | | | | | | | |
| TI  T2  T3 | Ref  -0.005(-0.12,0.11)  0.09 (-0.05,0.22) | Ref  -0.01(-0.11,0.09)  0.06(-0.06,0.18) | Ref  0.02(-0.05,0.09)  0.09(-0.003,0.17) | Ref  0.008(-0.02,0.03)  0.005 (-0.02,0.03) | Ref  -0.003(-0.13,0.13)  0.01(-0.11,0.14) | Ref  -0.5 (-1.0, 2.1)  1.3 (-0.6, 3.1) | Ref  0.4 (-0.6, 1.4)  1.0 (-0.1, 2.1) |
| **p-trend** | 0.219 | 0.315 | 0.044 | 0.726 | 0.851 | 0.172 | 0.088 |
| **Urban** | | | | | | | |
| TI  T2  T3 | Ref  0.03(-0.08,0.15)  **0.13(0.007, 0.26)** | Ref  0.008(-0.09,0.11)  0.11(-0.0003,0.22) | Ref  0.0006(-0.08,0.09)  -0.03(-0.08,0.14) | Ref  0.02(-0.005,0.05)  0.03(-0.00002, 0.06) | Ref  0.08(-0.04, 0.20)  **0.16(0.02,0.30)** | Ref  0.6 (-0.9, 2.2)  1.4 (-0.3, 3.2) | Ref  0.03 (-0.9, 1.0)  1.0 (-0.1, 2.1) |
| **p-trend** | 0.043 | 0.058 | 0.617 | 0.045 | 0.053 | 0.114 | 0.082 |
| **Migrant** | | | | | | | |
| TI  T2  T3 | Ref  0.02 (-0.11, 0.15)  0.08(-0.05, 0.22) | Ref  0.01(-0.10,0.13)  0.05(-0.07,0.17) | Ref  0.02(-0.05, 0.11)  0.02 (-0.07, 0.11) | Ref  -0.01 (-0.4,0.01)  **0.03(0.004,0.06)** | Ref  0.03(-0.08,0.14)  0.08(-0.03,0.18) | Ref  0.5 (-1.2, 2.2)  0.9 (-1.0, 2.8) | Ref  0.2 (-0.9, 1.3)  0.7 (-0.5, 1.7) |
| **p-trend** | 0.204 | 0.434 | 0.700 | 0.030 | 0.070 | 0.351 | 0.271 |
| p for interaction^#^ between animal food pattern and locations | 0.6009 | 0.5024 | 0.6827 | 0.1682 | 0.9722 | 0.9360 | 0.9417 |

*Robust standard error adjusted for age (continuous in years), sex (male/female), migration status (rural, urban, urban migrant), site(Lucknow, Nagpur, Hyderabad, Bangalore), SLI(continuous score), education (no formal education, primary school, secondary school and beyond secondary school), BMI(continuous in kg/m^2^), total energy(continuous in kcal/day), physical activity(continuous in totalMETS), tobacco(never, past, current), alcohol (never, past, current), use of any regular medication for chronic conditions such as diabetes, hypertension and/or food supplements (yes/no), sib-pair.

. **Analysis excluded known diabetics (n=486). #Wald test for interaction; Significant associations in bold

**Table S2** **(c):** Multivariable-adjusted linear associations* (beta co-efficient, 95% confidence interval) of ‘animal food’ pattern with cardio-metabolic risk factors by different standard of living (SLI) of the Indian Migration Study.

| Animal food pattern  tertiles | **Total cholesterol**  mmol/L | **LDL-C**  mmol/L | **Triglycerides**  mmol/L | **HDL-C**  mmol/L | **Fasting glucose****  mmol/L | **Systolic blood pressure** mm/Hg | **Diastolic blood pressure** mm/Hg |
| --- | --- | --- | --- | --- | --- | --- | --- |
| **Low** | | | | | |  |  |
| TI  T2  T3 | Ref  0.05(-0.06,0.16)  0.11(-0.01,0.24) | Ref  0.01(-0.08,0.11)  0.06(-0.06,0.17) | Ref  **0.07(0.005,0.14)**  **0.14 (0.06,0.22)** | Ref  0.01(-0.01,0.03)  0.02(-0.009,0.05) | Ref  -0.03(-0.12,0.05)  -0.09(-0.03,0.20) | Ref  1.5 (-0.04, 3.1)  1.8 (-0.03, 3.6) | Ref  0.8 (-0.2, 1.8)  1.0 (-0.1, 2.2) |
| **p-trend** | 0.074 | 0.331 | 0.001 | 0.174 | 0.152 | 0.052 | 0.075 |
| **Medium** | | | | | |  |  |
| TI  T2  T3 | Ref  -0.05(-0.17,0.06)  0.08(-0.05,0.21) | Ref  -0.04(-0.14,0.06)  0.06 (-0.05,0.17) | Ref  -0.01(-0.09,0.07)  0.004(-0.10,0.11) | Ref  -0.005(-0.03,0.02)  0.02(-0.003,0.05) | Ref  -0.02(-0.12,0.08)  0.08(-0.03,0.19) | Ref  -0.8 (-2.3, 0.7)  0.6 (-1.1, 2.3) | Ref  -0.8 (-1.8, 0.2)  0.5 (-0.6, 1.6) |
| **p-trend** | 0.244 | 0.292 | 0.946 | 0.089 | 0.345 | 0.520 | 0.367 |
| **High** | | | | | |  |  |
| TI  T2  T3 | Ref  0.03(-0.09,0.16)  0.12(-0.03,0.26) | Ref  0.03(-0.09,0.14)  0.11(-0.01,0.24) | Ref  -0.03(-0.12,0.07)  -0.01(-0.11,0.09) | Ref  0.02(-0.01,0.04)  0.02(-0.02,0.05) | Ref  0.14(-0.009,0.29)  0.13(-0.03,0.30) | Ref  1.3 (-0.5, 3.1)  1.7 (-0.4, 3.8) | Ref  0.7 (-0.4, 1.9)  **1.4 (0.1, 2.7)** |
| **p-trend** | 0.120 | 0.094 | 0.793 | 0.263 | 0.079 | 0.094 | 0.030 |
| p for interaction^#^ between animal food pattern and SLI | 0.5717 | 0.8696 | 0.1417 | 0.4774 | **0.0114** | 0.2811 | 0.1954 |

*Robust standard error adjusted for age (continuous in years), sex (male/female), migration status (rural, urban, urban migrant), site(Lucknow, Nagpur, Hyderabad, Bangalore), SLI(continuous score), education (no formal education, primary school, secondary school and beyond secondary school), BMI(continuous in kg/m^2^), total energy(continuous in kcal/day), physical activity(continuous in totalMETS), tobacco(never, past, current), alcohol (never, past, current), use of any regular medication for chronic conditions such as diabetes, hypertension and/or food supplements (yes/no), sib-pair.

. **Analysis excluded known diabetics (n=486). #Wald test for interaction; Significant associations in bold

**Table S3:** Multivariable adjusted* associations (beta co-efficient, 95% confidence interval) of daily consumption of individual animal food components in tertiles (fish /red meat/poultry/eggs) with cardio-metabolic risk factors of the Indian Migration Study participants (N=7067)

| **Animal food components**  **daily consumption in tertiles** | **Total cholesterol**  mmol/L | **LDL-C**  mmol/L | **Triglycerides**  mmol/L | **HDL-C**  mmol/L | **Fasting glucose****  mmol/L | **Systolic blood pressure** mm/Hg | **Diastolic blood pressure** mm/Hg |
| --- | --- | --- | --- | --- | --- | --- | --- |
| **Fish**  TI (0g/day)  T2 (0.1-6.2g/day)  T3 (6.3-1114.7g/day) | Ref  0.04(-0.03,0.11)  **0.13(0.05,0.2)** | Ref  0.03(-0.03,0.10)  **0.10(0.03,0.16)** | Ref  0.04(-0.01,0.09)  0.03(-0.02,0.08) | Ref  -0.0005(-0.02,0.02)  **0.02(0.002,0.04)** | Ref  0.01(-0.06,0.08)  0.04(-0.02,0.11) | Ref  0.06 (-0.9, 1.1)  0.5 (-0.5, 1.5) | Ref  -0.1 (-0.8, 0.5)  0.5 (-0.2, 1.1) |
| p-trend | 0.001 | 0.003 | 0.195 | 0.033 | 0.219 | 0.376 | 0.180 |
| **Red meat**  TI (0g/day)  T2 (0.1-12.6g/day)  T3 (12.7-629g/day) | Ref  0.06(-0.01,0.13)  **0.10(0.02,0.18)** | Ref  0.05(-0.01,0.11)  0.06(-0.008,0.13) | Ref  0.03(-0.02,0.07)  **0.08(0.02,0.14)** | Ref  -0.003(-0.02,0.01)  **0.02(0.0004,0.04)** | Ref  -0.003(-0.07,0.06)  0.02(-0.05,0.09) | Ref  0.7(-0.2, 1.6)  1.2 (1.0, 2.3) | Ref  0.4 (-0.2, 1.0)  0.9(0.2, 1.6) |
| p-trend | 0.012 | 0.071 | 0.012 | 0.072 | 0.613 | 0.028 | 0.009 |
| **Egg**  T1 (0g/day)  T2 (0.1-6.6g/day)  T3 (7.6-276g/day) | Ref  0.06(-0.01,0.13)  **0.15(0.07,0.22)** | Ref  0.04(-0.02,0.10)  **0.10(0.03,0.17)** | Ref  0.03(-0.02,0.08)  0.05(-0.002,0.11) | Ref  0.008(-0.007,0.02)  **0.03(0.01,0.05)** | Ref  0.05(-0.02,0.12)  0.07(-0.001,0.13) | Ref  0.7 (-0.3, 1.6)  1.0 (-0.04, 2.1) | Ref  0.8 (0.2, 1.4)  1.0 (0.3,1.6) |
| p-trend | <0.0001 | 0.004 | 0.063 | <0.0001 | 0.052 | 0.055 | 0.004 |
| **Poultry**  T1 (0g/day)  T2(0.1-14.7 g/day)  T3 (14.9-1194.6g/day**)** | Ref  0.07(-0.005,0.14)  **0.08(0.008,0.16)** | Ref  0.05(-0.02,0.11)  **0.07(0.007,0.14)** | Ref  0.03(-0.02,0.07)  0.03(-0.03,0.08) | Ref  0.008(-0.008,0.02)  0.01(-0.006,0.03) | Ref  0.008(-0.06,0.06)  0.03(-0.03,0.10) | Ref  0.3(-0.8, 1.3)  0.5(-0.5, 1.6) | Ref  0.1 (-0.5, 0.8)  0.5 (-0.1, 1.2) |
| p-trend | 0.027 | 0.028 | 0.309 | 0.182 | 0.345 | 0.299 | 0.106 |

*Robust standard error adjusted for age (continuous in years), sex (male/female), migration status (rural, urban, urban migrant), site(Lucknow, Nagpur, Hyderabad, Bangalore), SLI(continuous score), education (no formal education, primary school, secondary school and beyond secondary school), BMI(continuous in kg/m^2^), total energy(continuous in kcal/day), physical activity(continuous in totalMETS), tobacco(never, past, current), alcohol (never, past, current), use of any regular medication for chronic conditions such as diabetes, hypertension and/or food supplements (yes/no), sib-pair.

** Analysis excluded known diabetics (n=486); Significant associations in bold
